# Adolescent cognitive peculiarities and the sense of emerging adulthood

**DOI:** 10.1192/j.eurpsy.2022.1091

**Published:** 2022-09-01

**Authors:** O. Markish, S. Churbanova, O. Chesnokova

**Affiliations:** Lomonosov Moscow State University, Development Psychology Department, Moscow, Russian Federation

**Keywords:** Adolescents, emerging adulthood, self-critical cognitions, cognitive autonomy

## Abstract

**Introduction:**

The feeling of becoming an adult plays the role of central new mental formation reflecting the specificities of emotional experience and whole new mental state during adolescent development that is often misaligned with present day society attitude to adolescents. The study was conducted to explore the relationship linking cognitive peculiarities of senior adolescents and the sense of emerging adulthood

**Objectives:**

This research was conceptualized to explore the way cognitive development peculiarities affect the progress in solving tasks of the transition to adulthood from the point of view of objective growing-up and subjective assessment of feeling of becoming an adult.

**Methods:**

The study was based on Betensky’s Adolescent Window Triptych, Akimova’s Intelligence Test for Seniors, Landgarten’s Personality Collage, Sacks-Levy’s IST, Adolescent Social Self-Portrait Essay (D.B. Elkonin) and included 68 participants aged 15-17 years.

**Results:**

Self-criticism degree in cognition of elder adolescents has an effect on the progress in solving specific tasks of the transition to adulthood (such as high degree of maturity in intellectual activity (rs=.50; р=.002) and cognitive autonomy (rs=.36; р=.032), understanding importance of personal professional development (rs=.40; p=.059) and high value of having a family (rs=.39, p=.02). Also correlation regression analysis provides support for high correlation between self-esteem of personal autonomy, intellect (in solving tasks for conceptual thinking), emotional autonomy and social/moral maturity variables.

**Conclusions:**

It was confirmed that cognitive peculiarities of elder adolescents (such as academic intelligence, maturity in intellectual activity and cognitive autonomy) have an effect on the progress in solving specific tasks of the transition to adulthood.

**Disclosure:**

No significant relationships.

